# Pretreatment Plasma Circulating Tumor DNA RAS/BRAF Mutational Status in Refractory Metastatic Colorectal Cancer Patients Who Are Candidates for Anti-EGFR Rechallenge Therapy: A Pooled Analysis of the CAVE and VELO Clinical Trials

**DOI:** 10.3390/cancers15072117

**Published:** 2023-04-01

**Authors:** Davide Ciardiello, Stefania Napolitano, Vincenzo Famiglietti, Lucia Esposito, Vincenzo De Falco, Alessandra Di Liello, Antonio Avallone, Evaristo Maiello, Filippo Pietrantonio, Chiara Cremolini, Maria Giulia Zampino, Nicola Fazio, Teresa Troiani, Erika Martinelli, Fortunato Ciardiello, Giulia Martini

**Affiliations:** 1 Medical Oncology Unit, Department of Precision Medicine, University of Campania “Luigi Vanvitelli”, 80131 Naples, Italy; 2 Division of Gastrointestinal Medical Oncology and Neuroendocrine Tumors, European Institute of Oncology (IEO), IRCCS, 20141 Milan, Italy; 3 Experimental Clinical Abdominal Oncology Unit, Istituto Nazionale Tumori, IRCCS-Fondazione G. Pascale, 80131 Naples, Italy; 4 Oncology Unit, IRCCS Foundation Casa Sollievo della Sofferenza, 71013 San Giovanni Rotondo, Italy; 5 Medical Oncology Department, Fondazione IRCCS Istituto Nazionale dei Tumori, 20133 Milan, Italy; 6 Unit of Medical Oncology 2, University Hospital of Pisa, 56126 Pisa, Italy; 7 Department of Translational Research and New Technologies in Medicine and Surgery, University of Pisa, 56124 Pisa, Italy

**Keywords:** anti-EGFR drugs, rechallenge therapy, liquid biopsy, metastatic colorectal cancer

## Abstract

**Simple Summary:**

Rechallenge with epidermal growth factor (EGFR) inhibitors represents a promising therapeutic strategy in patients with refractory *RAS*/*BRAF* wild-type metastatic colorectal cancer (mCRC). The maximal benefit is observed in patients without resistance mutation at the baseline plasma circulating tumor DNA (ctDNA) evaluation. In the CAVE and VELO clinical trials, 1 out of 4 patients had ctDNA *RAS*/*BRAF* mutant disease at pretreatment liquid biopsy assessment. There was no direct association between the length of anti-EGFR drug-free interval and the presence of plasma ctDNA *RAS*/*BRAF* mutations at pretreatment liquid biopsy analysis. Interestingly, even the disappearance of mutant clones was time-dependent, and resistance mutations were found at liquid biopsy analysis in approximately 15% of patients after 18 or more months of anti-EGFR drug-free window. These results support the use of liquid biopsy to appropriately select amenable patients to EGFR inhibitor rechallenge.

**Abstract:**

Rechallenge with anti-EGFR drugs represents a promising strategy in refractory *RAS*/*BRAF* wild-type (WT) metastatic colorectal cancer (mCRC). We performed the pooled analysis of the CAVE and VELO studies to evaluate the percentage of patients with WT circulating tumor DNA (ctDNA) tumors and the association of mutational status with time from the last anti-EGFR drug administration. At baseline, 97/129 patients had *RAS*/*BRAF* WT plasma ctDNA, while 32/129 had *RAS*/*BRAF* mutated plasma ctDNA. Median anti-EGFR drug-free interval was 10.6 (CI 95%, 8.9–13.4) months in the plasma *RAS*/*BRAF* mutant group as compared to 13.0 (CI 95%, 11.1–16.6) months in *RAS*/*BRAF* WT group (*p* = 0.169). To investigate the time window of the *RAS*/*BRAF* mutant cancer cell clone disappearance, descriptive analysis using different time points was performed. No difference in the proportion of patients whose baseline plasma ctDNA was *RAS*/*BRAF* WT or mutated was found between 4 and 18 months since the last administration of anti-EGFR drugs. In contrast, 38/44 of patients with anti-EGFR drug-free interval of 18 months or more displayed a ctDNA *RAS*/*BRAF* WT status. Taken together, these results shows that the length of anti-EGFR free interval is not a sufficient criterion for patient selection, supporting the role of liquid biopsies for improving treatment efficacy.

## 1. Introduction

Better knowledge of the molecular biology of colorectal cancer (CRC) has radically changed the therapeutic scenario, allowing for the development of more effective therapies [1]. Treatment with anti-epidermal growth factor receptor (EGFR) drugs is a key therapeutic option for patients with *RAS* and *BRAF* wild-type (WT) tumors [2]. In this regard, the combination of anti-EGFR monoclonal antibodies and cetuximab or panitumumab with chemotherapy (5-fluorouracil and oxaliplatin or irinotecan) is associated with high response rates (60–70%) and durable disease control with a median progression-free survival (mPFS) of approximately 10–12 months [2,3,4]. Furthermore, there is a subset of patients with initial non-resectable liver metastases that achieve significant tumor shrinkage and could subsequently be candidates for radical surgery [5]. However, despite the initial response, most patients experience disease progression frequently related to acquired *RAS* mutations that occur in 30–40% of cases [2,3,4,5,6]. Unfortunately, after progression to first-line chemotherapy, subsequent lines of treatments are progressively less effective, with increasing toxicity due to accumulations of side effects [1,7,8,9,10]. For almost a decade, small tyrosine kinase inhibitor regorafenib and trifluridine/tipiracil were the only approved drugs in refractory mCRC [9,10]. Both drugs displayed limited clinical activity, with half of the patients that experienced disease progression at the first radiological assessment and with an overall survival of approximately 6–8 months. Moreover, tumor regression was anecdotical with an overall response rate (ORR) near to 1%. Thus, novel and more effective therapeutic options are required in the refractory setting. Increasing evidence supports that, in the absence of selective pressure by EGFR inhibitors, and during a subsequent treatment, there is a progressive reduction in anti-EGFR drug-resistant clones [11,12]. Parseghian and colleagues elegantly showed that *RAS* and *EGFR* mutant allele frequency decays exponentially after stopping anti-EGFR therapy with an estimated half-life of approximately 4 months [12].

Thus, after anti-EGFR drug-free intervals that allow for the clearance of resistant cancer cell clones, a rechallenge with EGFR inhibitors might represent an appealing therapeutic option in patients that benefit from prior anti-EGFR therapy [1,2,11]. On the basis of this rationale, our group and others showed that retreatment with anti-EGFR monoclonal antibodies (mAbs) alone or in combination with chemotherapy or immunotherapy has clinically relevant antitumor activity in a subset of patients with plasma circulating tumor DNA (ctDNA) *RAS*/*BRAF* wild-type (WT) tumors [13,14,15,16,17,18]. CRICKET was the first prospective Phase II trial that evaluated the activity of rechallenge with cetuximab and irinotecan as third-line treatment in mCRC [14]. In the overall population, the response rate was 21%. Interestingly, preplanned post hoc analysis showed that patients with baseline ctDNA *RAS*/*BRAF* WT tumors received the highest benefit. Despite the reduced number of included patients, the CRICKET study provided the first piece of evidence of a rechallenge with anti-EGFR in a molecularly selected population.

In the CAVE Phase II clinical trial, patients with pretreated *RAS* WT mCRC received cetuximab and avelumab as rechallenge therapy. The study was positive, with a median overall survival (mOS) of 11.6 months (95% CI, 8.4–14.8 months) and a median PFS (mPFS) of 3.6 months (95% CI, 3.2–4.1 months) in the intention to treat the population. Interestingly, mOS exceeded 17 months in the pretreatment plasma ctDNA *RAS*/*BRAF* WT population [15,16]. The VELO clinical trial was the first randomized Phase II study that assessed the role of rechallenge with panitumumab and trifluridine/tipiracil vs. trifluridine/tipiracil [18]. The primary endpoint was met, since the experimental treatment determined significant improvement in PFS compared to the control arm, with the maximal efficacy in patients without resistance mutation in EGFR pathway genes [18].

In the CHRONOS Phase II study, 27 molecularly selected patients received panitumumab as a later line of treatment [13]. Remarkably, 8/27 patients (30%) obtained a partial response (PR) with a disease control rate (DCR) of 63%. Taken together, these results suggest that there is a subset of patients that are still dependent on EGFR blockade even after initial progression. Therefore, the aforementioned data support the use of a liquid biopsy to select patients with plasma ctDNA *RAS*/*BRAF* WT tumors for anti-EGFR rechallenge therapy. However, there are several unsolved issues, including which the optimal anti-EGFR drug-free interval is before rechallenge, and if there is a correlation between the time of the last anti-EGFR drug administration and the persistence of *RAS*/*BRAF* mutations, in order to better identify patients without resistance mutations that are suitable for anti-EGFR rechallenge strategies.

Therefore, we performed the pooled analysis of the CAVE and VELO clinical trials, which have been the largest published anti-EGFR rechallenge therapy clinical trials.

## 2. Methods

CAVE (NCT04561336) was a single-arm, multicenter, Phase II trial evaluating the combination of cetuximab and anti-PD-L1 monoclonal antibody avelumab as rechallenge therapy in 77 patients with refractory *RAS* WT mCRC [15,16]. In particular, patients received a combination of avelumab (10 mg/kg every 2 weeks) and cetuximab (400 mg/m^2^ loading dose and subsequently 250 mg/m^2^ weekly) until disease progression or unacceptable side effects. The major inclusion criteria were that all patients must have had *RAS* WT mCRC, have obtained complete (CR) or partial response (PR) during first-line chemotherapy and an anti-EGFR drug, and have progressed and received at least second-line therapy after the failure of the first-line treatment. Moreover, an anti-EGFR drug-free interval of at least 4 months was required. The primary end point was OS, and the secondary end points were PFS, overall response rate (ORR), and safety. The study aimed to demonstrate an mOS of 11 months for the experimental arm as compared with the historical mOS of 8.0 months with trifluridine/tipiracil or regorafenib, which corresponds to an improvement of 37.5% in mOS. VELO (NCT05468892) was a multicenter, randomized, Phase II study investigating the role of combining panitumumab with trifluridine/tipiracil compared to trifluridine/tipiracil as a rechallenge in patients with *RAS* WT mCRC [18]. In total, 62 patients were randomized (1:1) to receive trifluridine/tipiracil (standard-of-care treatment, Arm A) or panitumumab and trifluridine/tipiracil (experimental treatment, Arm B) as third-line therapy. Panitumumab was administered as a 6 mg/kg intravenous infusion every 2 weeks of a 28-day cycle (Days 1 and 15). A 35 mg/m^2^ dose of trifluridine/tipiracil was orally administered twice daily, 5 days a week with 2 days of rest for 2 weeks, followed by a 14-day rest period. Treatment was administered in 28-day cycles until disease progression, unacceptable toxicity, the withdrawal of consent, or death due to any cause. The primary endpoint was PFS, and secondary endpoints were OS, ORR, and safety. The study was designed to have 80% power to detect a hazard ratio for PFS of 0.56 (44% reduction in risk) in the panitumumab and trifluridine/tipiracil arm as compared to the standard-of-care trifluridine/tipiracil arm, with a two-sided Type I error rate of 0.1.

The inclusion criteria were almost the same between the two studies, with the main difference being that, in the VELO trial, treatment was administered as third-line therapy in all patients, while a subgroup of patients enrolled in the CAVE study received rechallenge with cetuximab and avelumab as a later line of treatment.

For the present study, we selected patients enrolled in the two trials with available plasma samples for ctDNA analysis with a RT-PCR-based test (Idylla^TM^ Biocartis platform) at the baseline before trial treatment, and with complete clinical and pathologic data. The analysis was performed in the laboratory of the coordinating center (Department of Precision Medicine, Università degli Studi della Campania “Luigi Vanvitelli”, Napoli, Italy). At least 6 mL of whole blood was collected with the standard procedures with peripheral vein blood draw using Vacutainer^®^ with EDTA as the anticoagulant. Plasma was separated through two different centrifugation steps (the first at room temperature for 10 min at 1500× *g*, and the second at 2000× *g* for the same time and temperature). Plasma was stored at −80 °C until analysis in the Idylla^TM^ Biocartis platform. The complete process of the sample collection and processing, and the result analysis has been previously reported [19]. The detection limit for positivity for *KRAS* exons 2 and 3 or *BRAFV600E* mutations was >1%; for *KRAS* exon 4 and *NRAS* exons 2–4, it was >5%. The anti-EGFR free interval was defined as the time in months from the last EGFR inhibitor administration and enrollment in the CAVE and VELO studies, and ctDNA analysis. Correlation between anti-EGFR drug-free interval and mutational status was calculated with Mood’s median test. Moreover, patients were divided into different subgroups depending on the length of the anti-EGFR free interval (less than 6, between 6 and 11.99, 12 and 17.99, or more than 18 months). A descriptive evaluation of the frequency of plasma *RAS*/*BRAF* WT and mutant ctDNA in the different subgroups was performed. Statistical analyses were performed using the SPSS package (version 23, IBM).

## 3. Results

**Figure 1:**
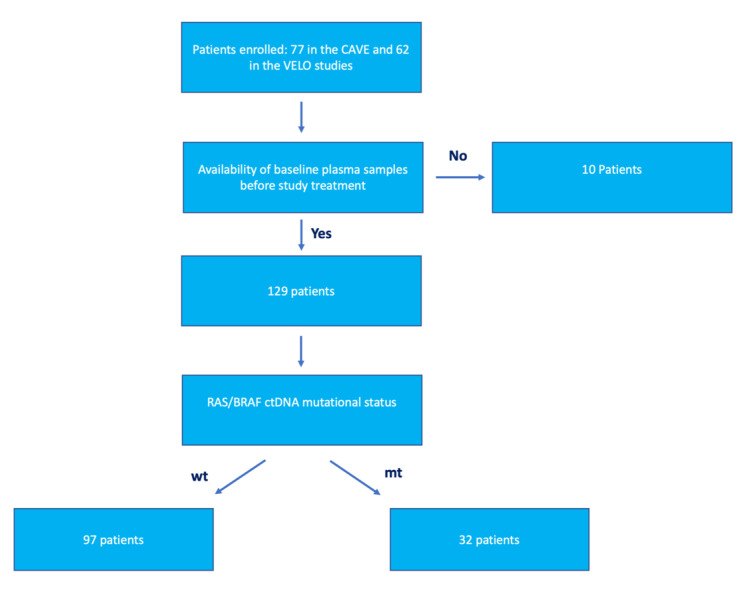
Patient selection flowchart. Of the 139 patients that had been enrolled in the CAVE and VELO clinical trials, 10 were excluded because their pretreatment plasma samples were not available. Of the 129 patients in the analysis, 97 had plasma circulating tumor DNA (ctDNA) *RAS*/*BRAF* wild type (WT), whereas 32 patients had plasma ctDNA *RAS*/*BRAF* mutations.

A total of 129 patients were included in the pooled analysis: 67/77 patients enrolled in the CAVE trial, and 62/62 patients from the VELO trial (Figure 1). In fact, only for 10/77 patients in CAVE study were pretreatment plasma samples not available for ctDNA evaluation.

**Figure 2:**
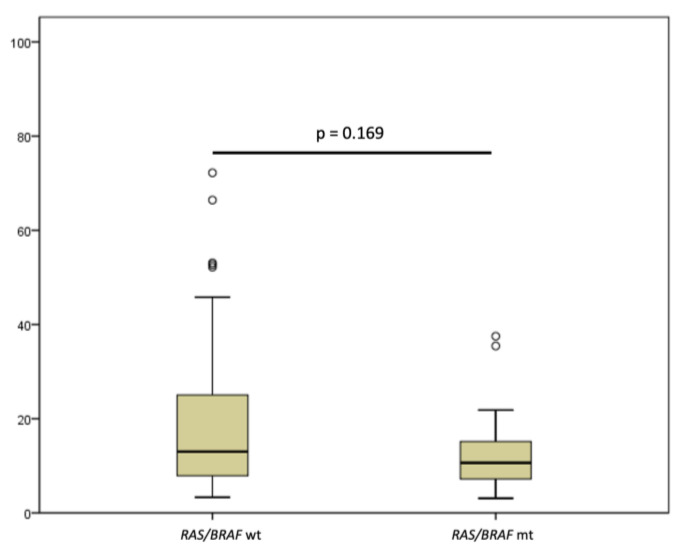
Association of median anti-epidermal growth factor (EGFR)-free interval and *RAS*/*BRAF* mutational status. Median anti-EGFR free interval was 10.6 months (CI 95% 8.95–13.4) in patients with *RAS*/*BRAF* mutant circulating tumor DNA (ctDNA), and 13.01 months (CI 95% 11.1–16.6) in patient with *RAS*/*BRAF* wild-type (WT) status (*p =* 0.169).

**Figure 3:**
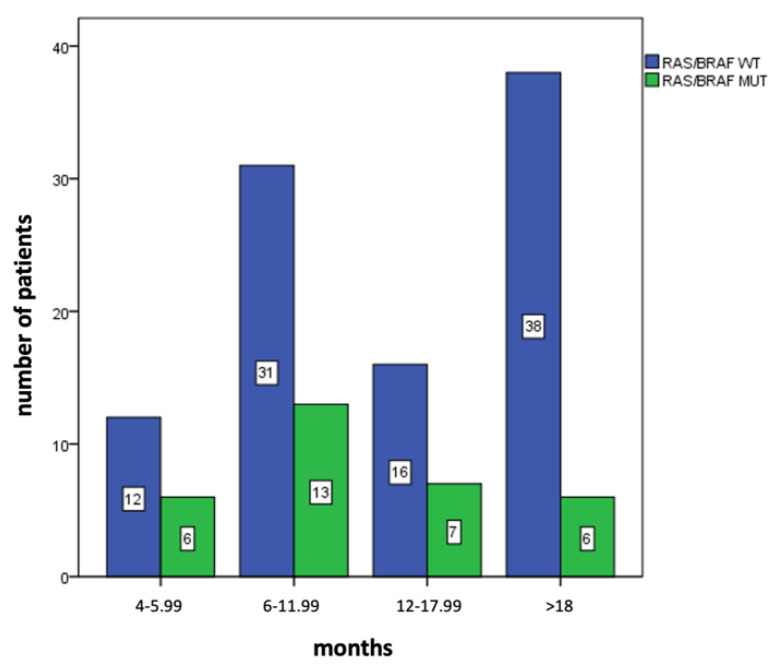
Descriptive analysis of *RAS*/*BRAF* mutational status in different subgroups depending on the length of the anti-EGFR free interval that was calculated as the time from the last anti-EGFR administration and baseline evaluation before study treatment in the CAVE and VELO clinical trials. WT: wild type; MUT: mutant.

The main patient characteristics are summarized in Table 1. Median age was 63 (range, 30–88) years; there were more male than female patients (56.6% vs. 43.4%); all patients had a good performance status (PS) (0 or 1 according to the ECOG scale). Most patients had the tumor located in the left colon or rectum (118/129, 91.5%) compared with the right colon (11/129, 8.5%). Primary tumor resection was performed in 92/129 (71.3%) patients. A high tumor burden with more than two different metastatic sites was observed in approximately one-third of the cases (47/129, 36.4%), with liver metastasis in half of the study population (67/129, 51.9%). The number of previous lines of treatment was 2 in most cases (113/129, 87.6%), with only 16 patients enrolled in the CAVE clinical trial who received rechallenge with cetuximab and avelumab in later lines of treatment. At the time of baseline assessment before anti-EGFR rechallenge therapy, by using the selection criteria of the two studies, plasma *RAS*/*BRAF* WT cDNA was found in approximately 3 out of 4 cases (97/129, 75.2%) via liquid biopsy analysis with the Idylla^TM^ Biocartis platform. *KRAS* mutations (30/32, 93.7%) were the most common gene alterations, *BRAF* mutations were observed in 2 patients, while an *NRAS* mutation was reported only in 1 case (Table 2). In this pooled analysis, no difference in the median time from the last administration of anti-EGFR drugs was observed in *RAS*/*BRAF* WT compared with mutant groups. In fact, the median anti-EGFR drug-free interval was 10.6 months (CI 95% 8.95–13.4) in patients with plasma *RAS*/*BRAF* mutant ctDNA, and 13.0 months (CI 95% 11.1–16.6, *p* = 0.169) in patient with plasma *RAS*/*BRAF* WT ctDNA (Figure 2). To further investigate if there was a potential correlation between the length of the anti-EGFR drug-free intervals and the disappearance of anti-EGFR cancer-resistant clones, we performed a descriptive analysis by using different time points from the last administration of anti-EGFR drugs in the first-line therapy (Figure 3). A reduced proportion of *RAS*/*BRAF* mutations was observed in the subgroup of patients (44/129) with very long anti-EGFR drug-free intervals, longer than 18 months. Of the patients, 38/44 (86.4%) in this subgroup displayed plasma *RAS*/*BRAF* WT ctDNA tumors; there was still a small subset of cases with ctDNA *RAS*/*BRAF* mutant tumors (6/44, 13.6%). These results indicate that the time from the last anti-EGFR drug treatment should not be considered the only parameter to select patients for anti-EGFR rechallenge strategies. Thus, a liquid biopsy should be performed before considering rechallenge with anti-EGFR-based therapies.

## 4. Discussion

The insurgence of mechanisms of secondary resistance is a limitation to the efficacy of anti-EGFR-based therapies [2,20,21,22,23,24,25,26,27]. Nevertheless, the complex and heterogeneous molecular landscape of resistance to anti-EGFR drugs is not a static condition, but rather dynamic and in continuous evolution depending on the use of anti-EGFR drugs, type of treatments, and previous lines of chemotherapy [26,27]. Interestingly, patients who had obtained a response with an anti-EGFR drug (panitumumab) as monotherapy were recently more likely to develop acquired resistance mutations (46%) as compared with those treated with panitumumab in combination with chemotherapy (9%) as frontline treatment [27]. Moreover, pretreatment-resistant subclonal mutant cancer cells rarely expand to become clonal at disease progression, remaining subclonal or even disappearing.

In this scenario, there could be room for an anti-EGFR drug rechallenge after a treatment holiday that allows for the clearance of resistant clones [11,12].

The present pooled analysis of the CAVE and VELO clinical trials represents, to our knowledge, the largest currently available dataset from prospective clinical studies. By using a RT-PCR-based (Idylla^TM^ Biocartis platform) we observed that 3 out of 4 patients (97/129, 75.2%) did not show *RAS/BRAF* mutation at the liquid biopsy analysis of plasma ctDNA before treatment. Thus, there is a significant number of patients that might benefit from a rechallenge with anti-EGFR drugs. These data are also potentially relevant since the Idylla^TM^ Biocartis could be used in clinical practice and allows for the detection of the main resistance mechanisms in a few hours [19]. However, this type of test has several limitations. The detection cut-off does not allow for identifying mutations with a low mutant allele fraction (MAF), which is a limit compared with more sensible tools such as digital-droplet PCR of NGS [28]. Nevertheless, the identification of the optimal MAF threshold and the real impact of *RAS* MAF < 5% on anti-EGFR drug response are still matters of debate [29]. Vidal and colleagues observed that the ultra-selection of patients with mCRC by increasing the detection threshold for *KRAS*/*NRAS*/*BRAF*/*PIK3CA* mutation from 5% to 1% by NGS was not correlated with improved outcomes. Therefore, the definition of the optimal limit detection for liquid biopsy analysis should be further investigated in larger prospective studies.

Another intrinsic limitation of this RT-PCR-based test is the possibility of detecting only hot-spot mutations and thereby missing other potential mechanisms of resistance. Despite these limitations, the reported results here are in line with findings from other groups [13,30]. In the CHRONOS trial, 16 out of 52 (31%) patients that were candidates for an anti-EGFR rechallenge with panitumumab had pretreatment plasma *RAS*/*BRAF* mutated ctDNA at digital-droplet PCR analysis. The PARERE (NCT04787341) study is a prospective, multicenter, randomized Phase II trial investigating a rechallenge with panitumumab compared with regorafenib and the inverse sequence in 214 patients with chemorefractory mCRC with baseline ctDNA *RAS*/*BRAF* WT tumors. A preliminary analysis of the PARERE study was presented at the ESMO World Congress on Gastrointestinal Cancer 2022 [30]. Of the 101 patients that entered the screening phase, the pretreatment plasma samples of 90 patients were evaluated for ctDNA analysis. In 25/90 (28%) of those patients, *KRAS*/*NRAS* or *BRAFV600E* mutations were detected. Our group is currently conducting a large and randomized Phase II study (CAVE 2 trial) investigating the combination of cetuximab and avelumab compared with cetuximab as a rechallenge strategy in 173 patients with refractory mCRC [31]. During the screening phase, baseline plasma samples were analyzed for ctDNA according to the NGS (FoundationOne liquid test) to identify patients without resistance mutations to be enrolled in the study.

By increasing the time from the last administration of anti-EGFR drugs, a progressive reduction in resistant cancer cell clones was proposed [11,12]. In the pooled CAVE and VELO analysis, there was no difference in the median anti-EGFR free intervals in patients with pretreatment plasma *RAS*/*BRAF* WT ctDNA as compared with patients with *RAS*/*BRAF*-mutated ctDNA. In the subgroups of patients with an anti-EGFR free interval of. less than 18 months, no major differences in the proportion of *RAS*/*BRAF* ctDNA mutant vs. wild-type tumors were reported. However, a relatively long anti-EGFR drug-free period of more than 18 months was associated with the reduced probability of having mutant tumors (38/44 patients displayed *RAS/BRAF* WT tumors). Nevertheless, approximately 14% of these patients still had *RAS*/*BRAF* mutant tumors, suggesting that the temporal criteria are not sufficient by themselves for patient selection.

## 5. Conclusions

Rechallenge with anti-EGFR drugs for patients with refractory mCRC is a promising option for chemo-refractory *RAS*/*BRAF* wt mCRC. Robust translational studies have proven that the half-life of resistant clones is approximately 4 months after the cessation of anti-EGFR administration. The results of the pooled analysis of the CAVE and VELO clinical trials support the use of defined clinical criteria and pretreatment evaluation of *RAS/BRAF* ctDNA mutational status with liquid biopsies for the appropriate selection of patients that could effectively benefit from anti-EGFR rechallenge therapy in the continuum of care of mCRC. Nevertheless, there are some open questions. One of the main issues is the identification of better assays for liquid biopsy NGS vs. PCR tests. Furthermore, the availability of a liquid-biopsy test could be a major problem outside referral centers. Lastly, the optimal cut-off for *RAS/BRAF* mutations in the liquid biopsy has not yet been defined and is debated. Studies are ongoing to clarify the role of anti-EGFR rechallenge in the continuum of care of mCRC [2,11,30,31].

## Figures and Tables

**Table 1:** Characteristics of the study population. F: female; M: male; ctDNA: circulating tumor DNA; WT: wild type; MT: mutant.

Characteristics	N = 129
Age	
Years (median)	63 (30–88)
Sex	
F	56 (43.4%)
M	73 (56.6%)
Performance status	
0	86 (66.7%)
1	43 (33.3%)
Tumor location	
Left colon and rectum	118 (91.5%)
Right colon	11 (8.5%)
Primary tumor resection	
Yes	92 (71.3%)
No	37 (28.7%)
Number of metastatic sites	
<3	82 (63.6%)
≥3	47 (36.4%)
Liver metastasis	
Yes	67 (51.9%)
No	62 (48.1%)
Number of previous lines of treatment	
2	113 (87.6%)
3	8 (6.2%)
4	4 (3.1%)
5	2 (1.6%)
6	2 (1.6%)
Baseline ctDNA	
*RAS*/*BRAF* wt	97(75.2%)
*RAS*/*BRAF* mt	32 (24.8%)

**Table 2:** Type of *KRAS*/*NRAS*/*BRAF* mutations that were observed during pretreatment plasma liquid biopsy analysis by using the Idylla^TM^ Biocartis platform.

Type of RAS/BRAF Mutation	Number of Patients (32)
BRAF V600E	1
KRAS A146P	2
KRAS A146PT	1
KRAS A146PV	1
KRAS G12A	3
KRAS G12C	2
KRAS G12D	5
KRAS G12S	2
KRAS G12V	2
KRAS G13D	3
KRAS Q61H	6
KRAS Q61H and BRAF V600E	1
KRAS Q61R	1
NRAS G13D	1
KRAS Q61L	1

## Data Availability

Data are available in anonymized form upon reasonable request.
